# *icaR* and *icaT* are Ancient Chromosome Genes Encoding Substrates of the Type III Secretion Apparatus in Shigella flexneri

**DOI:** 10.1128/msphere.00115-22

**Published:** 2022-05-02

**Authors:** Navoun Silué, François-Xavier Campbell-Valois

**Affiliations:** a Host-Microbe Interactions Laboratory, Center for Chemical and Synthetic Biology, Department of Chemistry and Biomolecular Sciences, University of Ottawa, Ottawa, Ontario, Canada; b Centre for Infection, Immunity, and Inflammation, University of Ottawa, Ottawa, Ontario, Canada; c Department of Biochemistry, Microbiology and Immunology, University of Ottawa, Ottawa, Ontario, Canada; University of Iowa

**Keywords:** *Escherichia coli*, *Shigella*, type III secretion system, phylogeny, transcription regulation

## Abstract

*Shigella* is an Escherichia coli pathovar that colonizes the cytosol of mucosal cells in the human large intestine. To do this, *Shigella* uses a Type III Secretion Apparatus (T3SA) to translocate several proteins into host cells. The T3SA and its substrates are encoded by genes of the virulence plasmid pINV or by chromosomal genes derived thereof. We recently discovered two chromosomal genes, which seem unrelated to pINV, although they are activated by MxiE and IpgC similarly to some of the canonical substrates of the T3SA. Here, we showed that the production of the corresponding proteins depended on the conservation of a MxiE box in their cognate promoters. Furthermore, both proteins were secreted by the T3SA in a chaperone-independent manner through the recognition of their respective amino-terminal secretion signal. Based on these observations, we named these new genes *icaR* and *icaT*, which stand for invasion chromosome antigen with homology for a transcriptional regulator and a transposase, respectively. *icaR* and *icaT* have orthologs in commensal and pathogenic E. coli strains belonging mainly to phylogroups A, B1, D and E. Finally, we demonstrated that *icaR* and *icaT* orthologs could be activated by the coproduction of IpgC and MxiE in strains MG1655 K-12 (phylogroup A) and O157:H7 ATCC 43888 (phylogroup E). In contrast, the coproduction of EivF and YgeG, which are homologs of MxiE and IpgC in the E. coli T3SS 2 (ETT2), failed to activate *icaR* and *icaT*.

**IMPORTANCE** *icaR* and *icaT* are the latest members of the MxiE regulon discovered in the chromosome. The proteins IcaR and IcaT, albeit produced in small amounts, are nonetheless secreted by the T3SA comparably to canonical substrates. The high occurrence of *icaR* and *icaT* in phylogroups A, B1, D, and E coupled with their widespread absence in their B2 counterparts agree with the consensus E. coli phylogeny. The widespread conservation of the MxiE box among *icaR* and *icaT* orthologs supports the notion that both genes had already undergone coevolution with transcriptional activators *ipgC* and *mxiE*- harbored in pINV or a relative- in the last common ancestor of *Shigella* and of E. coli from phylogroups A, B1, D, and E. The possibility that *icaR* and *icaT* may contribute to *Shigella* pathogenesis cannot be excluded, although some of their characteristics suggest they are fossil genes.

## INTRODUCTION

*Shigella* is an Escherichia coli pathovar infecting the large intestine of humans and one of the major causes of diarrheal diseases. Its pathogenesis is characterized by the invasion of mucosal epithelial cells and also immune cells, particularly macrophages residing in the *lamina propria* (1). The type III secretion system (T3SS) of *Shigella* is essential to the invasion of the cytosol of host cells and the resistance to cell-autonomous immunity (2).

The T3SS is chiefly composed of genes required for the assembly of the type III secretion apparatus (T3SA), a megadalton protein complex resembling a syringe (3, 4), which can distinguish its substrates among thousands of cytosolic proteins and secrete them in an orderly fashion. Early substrates are components of the needle. Intermediate substrates are translocators, which form the translocon that allows the transfer of effectors from the T3SA needle into the cytosol. Finally, the late substrate consists of effectors that hijack host cell processes (5). Besides, the T3SA and its substrates, the T3SS also include chaperones that are essential for the stability and secretion of some substrates and transcriptional regulators that control the expression of the T3SS components (5).

The T3SS of *Shigella* spp. and related enteroinvasive Escherichia coli (EIEC) is encoded in the virulence plasmid pINV (6), which is named differently in each strain (e.g., pWR100 in strain M90T). The T3SA of Shigella flexneri strain M90T has currently 35 known or suspected protein substrates (7). In *Shigella*, late substrates form two main classes (5). The late substrate A are stored effectors, which must bind to one of three chaperones IpgA, IpgE or Spa15 during their cytosolic storage in order to be optimally secreted upon T3SA activation. The late substrates B are effectors that are produced at significant levels only when T3SA are active (5). They are thus secreted in a chaperone-independent manner as soon as they are produced (8). The expression of genes encoding late substrate B depends on the transcription activator MxiE and the co-activator IpgC. These proteins form a complex that activates the transcription of their target genes through the binding of a MxiE box located at the 5′ end of the corresponding promoters (5). The formation of the MxiE-IpgC complex peaks when the cytosolic store of their inhibitors OspD1 and IpaBC is depleted through their T3SA-mediated secretion, thereby coupling the expression of the MxiE regulon to the activity of the T3SA (5). *In vitro*, it is possible to induce the secretion of wild-type (WT) cells with the dye Congo red (CR). Alternatively, one can study constitutive secretion of mutants that have defects in the *ipaD* or *ipaB* genes, which encode proteins forming the tip complex that acts as a repressor of constitutive secretion in the WT (5). The transcription activation mediated by the T3SA is so exquisitely well controlled that the coupling of a MxiE-regulated promoter to a fast-maturing variant of the GFP afforded a reporter highlighting bacteria that are actively secreting in tissue culture cells or animal tissues (9, 10). Genes that are activated by MxiE-IpgC encode effectors such as OspF, VirA, and the IpaH family (11). These genes are located in pINV, with the notable exception of the 7 *ipaH* genes located in the chromosome (11, 12).

Recently, we exhaustively probed the genome of S. flexneri str. M90T to uncover unknown members of the MxiE regulon by comparing the transcriptome of WT (inactive T3SA) and Δ*ipaD* (constitutively active T3SA) using RNA sequencing (RNAseq). This led to the identification of two new chromosome genes, which transcription regulation resembled those of canonical members of the MxiE regulon, albeit being expressed one order of magnitude lower (5, 13). These genes were annotated as coding, but because the properties of the corresponding proteins were unknown, we temporarily named them gem1 and gem3. Their transcription start sites (2,469,433 and 4,386,701, respectively) are at least 200 kbp from other chromosome-encoded T3SS genes (14). In addition, gem1 and gem3 possess a consensus MxiE box, suggesting that their transcription regulation is autonomous (13). Their GC content is significantly lower than the chromosome, suggesting they were acquired by horizontal gene transfer. Nevertheless, both genes have no sequence homology with pINV. Interestingly, orthologs of both genes are found in pathogenic and nonpathogenic E. coli strains. The primary structure of the protein encoded by gem1 had low homology with transposases associated with insertion sequences (IS). Its homolog in E. coli K-12 is annotated *yfdF* and encodes a hypothetical protein. Instead, the primary structure of the protein encoded by gem3 is annotated as DNA binding protein. Indeed, the Predict Protein server gave a high DNA binding score to segments of this protein located between residues 40 and 160 (15). The primary structure of this protein was highly similar to the N-terminal region of the YjgL hypothetical protein of E. coli K-12. The introduction of an IS sequence at the 3′ end of gem3, however, truncated its coding sequence in *Shigella*. Here, we show that the production of the proteins encoded by these genes depends on the MxiE box in their respective promoter and that both proteins are substrates of the T3SA. By analogy with the invasion plasmid antigen genes (*ipa*; e.g., *ipaH*s), we renamed them *icaT* (gem1) and *icaR* (gem3), which stands for *i*nvasion *c*hromosome *a*ntigen with homology for a *T*ransposase and transcription *R*egulator, respectively. Finally, we also demonstrate that E. coli orthologs of *icaR* and *icaT* could be activated by the coproduction of MxiE and IpgC.

## RESULTS

### The production of IcaR and IcaT required MxiE, IpgC, and a MxiE box.

**FIG 1:**
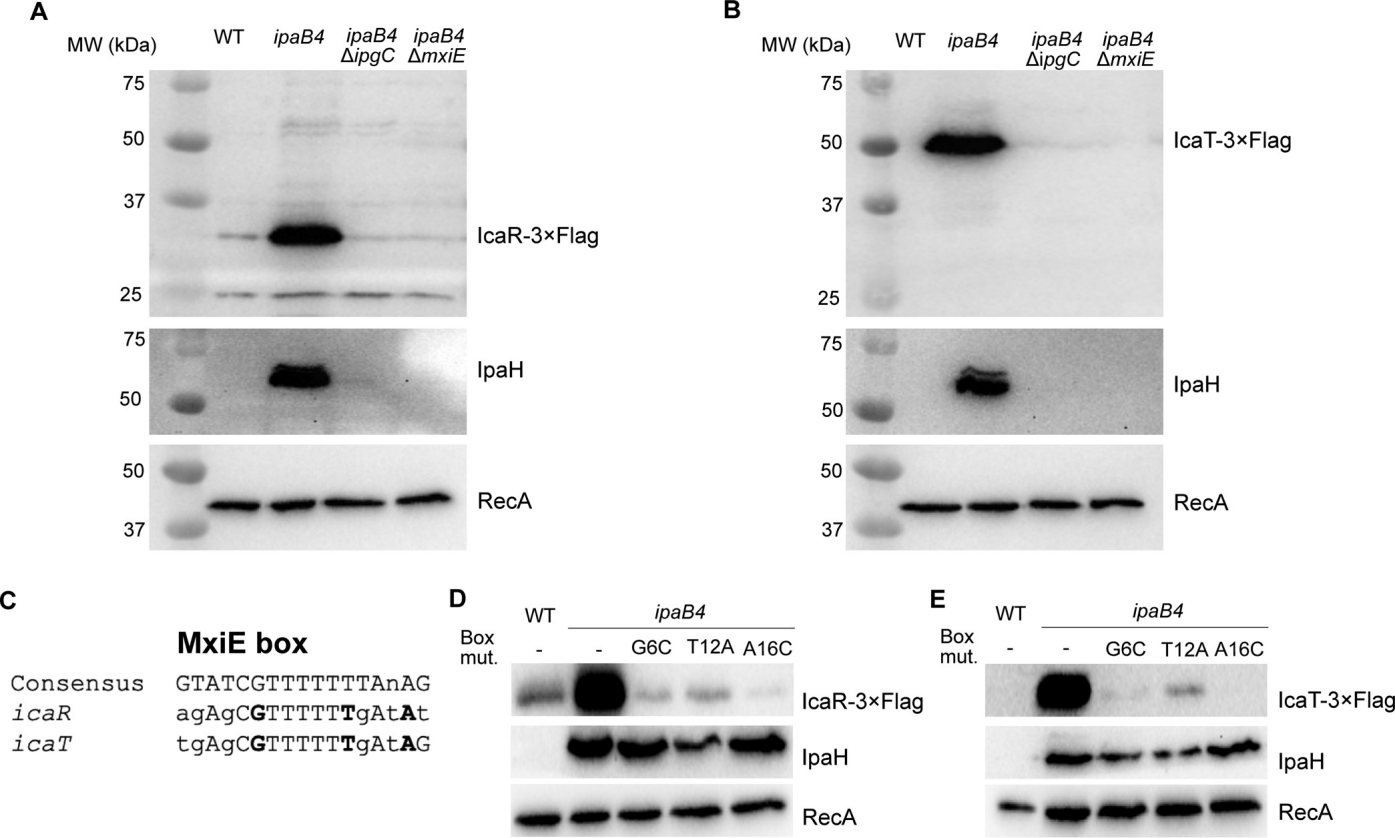
MxiE and IpgC are required to produce IcaR and IcaT. (A and B) Detection of IcaR and IcaT by immunoblotting with a FLAG-antibody in the total cell lysate (TCL) of the indicated S. flexneri M90T strains harboring plasmid-born *icaR* and *icaT* placed under the control of their endogenous promoters and grown in TSB at 37°C. IpaH and RecA, for which production is dependent and independent of MxiE and IpgC, respectively, were used as controls. (C) Alignment of *icaR* and *icaT* putative MxiE boxes with the consensus MxiE box. The nucleotides in bold are mutated to validate the putative MxiE boxes. (D and E) Detection of IcaR and IcaT, using the method described above, in strains harboring plasmid-borne *icaR* and *icaT* in which the indicated mutations were inserted into their MxiE box. In the experimental conditions used here the MxiE regulon is activated in *ipaB4* and its derivatives, but not in the WT strain. The results are representative of three independent experiments.

We previously showed that the transcription of *icaR* and *icaT* was upregulated in a MxiE-dependent manner. Typically, genes belonging to the MxiE regulon are expressed to high levels only when T3SA were active (5, 13). This active state allows the association of an upstream promoter element name the MxiE box with a MxiE-IpgC complex, which favors the initiation of transcription. To verify whether this model applied to *icaR* and *icaT*, we analyzed the production of the corresponding proteins. To do this, we subcloned the promoter region and the coding sequence of *icaR* and *icaT* into a promoterless plasmid, which allowed the detection of their cognate proteins with a 3×FLAG epitope. This plasmid was then transformed into WT and *ipaB4* or its derivatives *ipaB4* Δ*mxiE* and *ipaB4* Δ*ipgC*. The *ipaB4* strains harbor a nonfunctional allele of *ipaB*, making these cells constitutively secrete *in vitro*, in contrast with the WT, which is not secreting under the same conditions. The Western blot (WB) of the total cell lysate (TCL) confirmed that IcaR and IcaT, similarly to the endogenous IpaH proteins (IpaHs) were produced by constitutively secreting *ipaB4* in an *ipgC* and *mxiE*-dependent manner, whereas they were not produced in the WT, and RecA production was invariable in all strains (Fig. 1A and B). IcaR-3×FLAG migrated according to its expected MW of 31 kDa. IcaT-3×FLAG migrated around 50 kDa, although its expected MW was 44 kDa. It is noteworthy that the pI of IcaT is acidic (5.1), which is notorious for reducing electrophoretic mobility. In addition, we identified a putative MxiE box within the promoter of *icaR* and *icaT* that aligned well with the consensus MxiE box (Fig. 1C). Guided by this alignment (11), we introduced point mutations at key positions of the MxiE box of *icaT* and *icaR*. As expected, all mutations reduced the production of plasmid-borne IcaR and IcaT in *ipaB4*, whereas RecA and IpaHs produced from their intact chromosome loci were invariable. In the case of *icaR*, we noted the presence of a second putative MxiE box partly overlapping with the first one. Mutations of this box, however, did not impact the production of IcaR, suggesting it was not involved in the regulation of the promoter (unpublished data). Taken together, these data indicated that *icaRp* and *icaTp* allowed the expression of their cognate proteins in response to secretion as previously shown for other MxiE-regulated genes.

### IcaR and IcaT were secreted in a T3SA-dependent manner.

**FIG 2:**
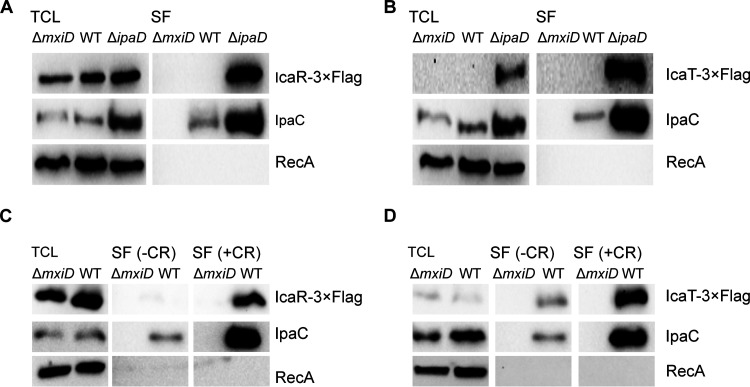
IcaR and IcaT are secreted in a T3SA-dependent fashion. (A and B) Detection of IcaR and IcaT by immunoblotting with a FLAG-antibody in the total cell lysate (TCL) and the secreted fraction (SF) of the indicated S. flexneri 5a str. M90T strains harboring plasmid-born *icaR* and *icaT* placed under the control of the *lacZ* promoter and grown in TSB at 37°C. IpaC, which is a T3SA substrate, and RecA, a housekeeping protein, were used as controls. (C and D) Detection of IcaR and IcaT by immunoblotting with a FLAG-antibody in the TCL and SF, as described above, and in the presence or absence of the secretion inducer Congo red (CR). Δ*mxiD* is T3SA-deficient. T3SA of the WT strain can be activated by CR whereas they are constitutively active in Δ*ipaD*. The results are representative of three independent experiments.

Because the proteins encoded by canonical MxiE-regulated genes are substrates of the T3SA (5), we wondered whether the same was true for *icaR* and *icaT*. To test this hypothesis, we used two well-validated approaches that measure the secretion of T3SA substrates in the extracellular medium. The first approach consists in the comparison of the TCL and a secreted fraction (SF) of the *ΔmxiD* (T3SA-negative), WT (T3SA were inactive in the absence of inducer), and Δ*ipaD* (T3SA were constitutively active, as in *ipaB4*) (16). The second approach compared TCL and SF of Δ*mxiD* and WT in the presence or absence of Congo red (CR), a chemical inducer of secretion, using a classical assay that measures the secretion of substrates that were stored in the cytosol (17). Because the activity of MxiE-regulated promoters was low in Δ*mxiD* and the WT in the absence of CR, the use of endogenous promoters would lead to a diverging level of expression of the various strains required to perform these assays. To circumvent this issue, the endogenous promoters were replaced with *lacZ*p, which is a constitutive promoter in *Shigella*, and the expression of IcaR and IcaT were probed by WB as described in Fig. 1. We collected the TCL and SF of Δ*mxiD*, WT, and Δ*ipaD* strains in the absence or presence of CR (Fig. 2). In the absence of CR, IcaR was invariable in the TCL of the three strains (Fig. 2A). As expected, the SF of Δ*ipaD* contained a greater amount of IcaR and the positive-control IpaC than those of WT and Δ*mxiD* cells. To detect their expression from their chromosome locus, we raised polyclonal antibodies against these proteins. The serum raised against IcaT was unreactive against the purified recombinant protein. Fortunately, the serum raised against IcaR was more promising. Indeed, a band with the predicted MW of 27 kDa was specifically observed in the TCL of Δ*ipaD* using this serum (Fig. S1A). The Δ*ipaD* harboring the plasmid with *lacZ*p::*icaR*-3×FLAG used in Fig. 2 yielded a second band with an apparent molecular weight of 31 kDa as expected, thus demonstrating that this serum recognized IcaR. The intensity of bands at 27 kDa and 31 kDa suggests the recombinant protein was produced comparably to the endogenous protein. Finally, immunoblotting of the SF revealed that endogenous IcaR was indeed secreted in a T3SA-dependent manner (Fig. S1B).

10.1128/msphere.00115-22.1 FIG S1 IcaR produced from its endogenous locus is secreted in a T3SA-dependent fashion. (A) Detection of endogenous and recombinant IcaR by immunoblotting with a polyclonal antibody in the total cell lysate of the indicated S. flexneri 5a str. M90T derivatives grown in TSB at 37°C. pNS9 harbors *lacZ*p::*icaR*-3×FLAG. Hence, Δ*ipaD* pNS9 displayed two bands corresponding to the endogenous and recombinant IcaR at 27 kDa and 31 kDa, respectively. Protein X with an apparent MW of 20 kDa cross-reacted with the antibody, but it was easily distinguished by its mass and its constitutive production. (B) Detection of endogenous IcaR by immunoblotting in the total cell lysate and the secreted fraction of the indicated S. flexneri 5a str. M90T derivatives grown in TSB at 37°C. The endogenous IcaR was detected with a polyclonal antibody raised against its residues 143 to 158. Download FIG S1, EPS file, 1.6 MB. Copyright © 2022 Silué and Campbell-Valois. 2022 Silué and Campbell-Valois. https://creativecommons.org/licenses/by/4.0/ This content is distributed under the terms of the Creative Commons Attribution 4.0 International license.

Furthermore, using the classical CR assay (17), the secretion of IcaR was detected in the WT, but not in Δ*mxiD* (Fig. 2B). Whereas IcaT was detectable in the TCL and secreted fraction of Δ*ipaD* as expected (Fig. 2C), it was undetectable in both the TCL and a secreted fraction of WT and Δ*mxiD.* Because IcaT was well expressed in the constitutive secretion state established in Δ*ipaD*, we hypothesized that IcaT was unstable in the cytosol of Δ*mxiD* and WT, thus precluding the detection of its secretion using the classical CR assay, which measures the secretion of stored effectors (data not shown). We reasoned that WT cells grown in the presence of CR should be able to secrete the protein before it might be degraded in the cytosol. Using this protocol, the level of IcaT in the TCL of the WT strain was increased, and this protein as well as IpaC were detected in the secreted fraction of the WT strain but not in that of the Δ*mxiD* strain (Fig. 2D).

**FIG 3:**
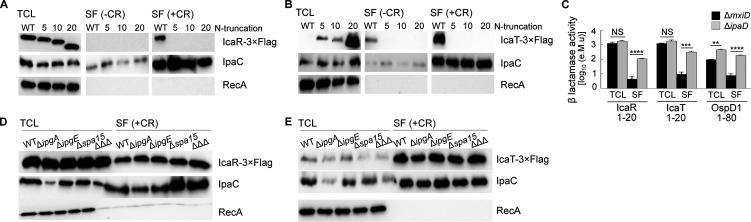
The secretion of IcaR and IcaT depended on their amino terminus and was chaperone-independent. (A and B) Detection of IcaR and IcaT N-terminal truncation mutants (0, 5, 10, or 20 residues truncation) by immunoblotting with a FLAG-antibody in the total cell lysate (TCL) and the secreted fraction (SF) of the indicated S. flexneri 5a str. M90T strains harboring plasmid-born *icaR* and *icaT* placed under the control of the *lacZ* promoter and grown in TSB at 37°C in the presence or absence of the secretion inducer Congo red. IpaC, which is a T3SA substrate, and RecA, a housekeeping protein, were used as controls. (C) Nitrocefin assays performed on the TCL and SF of Δ*mxiD and* Δ*ipaD* strains producing IcaR (residues 1 to 20), IcaT (residues 1 to 20) or OspD1 (residues 1 to 80) N terminus and their corresponding deletion mutants fused to *bla*_TEM3_ M182T. Student’s *t* tests for unpaired data with a 95% confidence interval are shown., NS, not significant; *, *P* ≤ 0.05; **, *P* ≤ 0.01; ***, *P* ≤ 0.001; ****, *P* ≤ 0.0001. (D and E) Detection of full-length IcaR and IcaT by immunoblotting with a FLAG-antibody in the TCL and SF of S. flexneri 2a str. 2457T harboring plasmid born *icaR* and *icaT* under the control of the *lacZ* promoter and cultivated as above. The ΔΔΔ is a triple-knockout strain devoid of the three chaperones (e.g., IpgA, IpgE, and Spa15) required for the secretion of some of the late substrates in *Shigella*. The results are representative of three independent experiments.

We wondered whether the secretion signal of both IcaR and IcaT was located at the amino-terminal end, as observed for bona fide T3SA substrates. Indeed, deletion of 5, 10, or 20 residues at their N terminus abrogated CR-induced secretion and constitutive secretion of Δ*ipaD* (Fig. 3A and B and unpublished data). The Δ20 IcaT was expressed to a higher level than the full-length IcaT. This suggests the N terminus, which was predicted by Alpha fold and RoseTTAFold to be a random coil (18, 19), destabilized the protein, likely rendering it susceptible to cytosolic proteases. Next, we asked whether the N terminus of IcaR and IcaT were sufficient to induce the secretion. To test this, we measured the nitrocefin hydrolysis activity of the TCL and SF of Δ*mxiD* and Δ*ipaD* strains producing cytosolic *bla*_TEM3_ M182T as C-terminal fusion to residues 1 to 20 of IcaR and IcaT. First, the TCL containing IcaR and IcaT fusions yielded comparable nitrocefin hydrolysis activity, indicating their production in Δ*mxiD* and Δ*ipaD* were similar, and stemming from this, that difference in the SF would correlate with the respective secretion level of the fusion proteins (Fig. 3C). Second, nitrocefin hydrolysis was detected in the SF of Δ*ipaD*, but not in Δ*mxiD*, suggesting the secretion of *bla*_TEM3_ mediated by residues 1 to 20 of IcaR and IcaT was T3SA-dependent. In addition, the efficacy of the secretion mediated by residues 1 to 20 of IcaR and IcaT was comparable to residues 1 to 80 of OspD1 (20), a validated late substrate A of the T3SA (5). Because IcaR and IcaT share the same type of N terminus and transcription regulation as the late substrates B, we reasoned that their secretion should be likewise independent of T3SS chaperones. To test this hypothesis, we used the CR assay on WT S. flexneri 2a str. 2457T and isogenic strains Δ*ipgA*, Δ*ipgE*, Δ*spa15,* and the corresponding triple-knockout strain ΔΔΔ (8). The amount of IcaR and IcaT in the secreted fraction of these strains was equivalent to the WT, suggesting their secretion was indeed chaperone-independent (Fig. 3D and E). Thus, these data support the notion that IcaR and IcaT were late substrates B of the T3SA in *Shigella* (5).

### Orthologs of *icaR* and *icaT* were found in several phylogroups of E. coli.

Using BLAST, we identified orthologs of *icaR* and *icaT* in several *Shigella* subgroups and E. coli phylogroups (Table S1). Because E. coli strains abound in databases, we performed another BLAST search that excluded E. coli and *Shigella*, to probe the occurrence of *icaR* and *icaT* in other species. We found that *icaT* was only present in two poorly studied Salmonella spp. In contrast, *icaR* also occurred frequently in Escherichia albertii. In addition, *icaR* and *icaT* seemed absent in Escherichia marmotae and Escherichia fergusonii. Given the uncertainties in the phylogenetic relationships between species in the Escherichia genus (21), we decided to focus on E. coli. Indeed, the locus of *icaR* and *icaT* were conserved across E. coli (Fig. S2), suggesting these genes appeared in this lineage through two independent chromosomal insertion events in the vicinity of *argF* and *fadL*, respectively. The locus of *icaT* was relatively well-preserved, whereas the locus of *icaR* is disrupted to variable extents by IS that were phylogroup or even strain-specific. The disruption of *icaR* culminates in *Shigella* with a large 3′ deletion (Fig. S2). Indeed, a nucleotide BLAST (BLASTn) with the full-length *icaR* from MG1655 yielded a single hit with 100% query coverage (S. flexneri str. C32), suggesting that *icaR* truncation is widespread in *Shigella*. The size of the 3′ deletion in *icaR* varies in S. flexneri, S. sonnei, and S. boydii, suggesting that IS landed on this locus on several independent occasions (Fig. S2). To quantify the disruption of these genes, we counted the occurrence of integral *icaR* and *icaT* using BLASTn within each *Shigella* subgroups using M90T orthologs as queries (Table S2). Overall, *icaR* and *icaT* appeared severely disrupted in S. dysenteriae, whereas they were less disrupted in the other subgroups, particularly in S. flexneri in which a large proportion of strain seems to possess integral *icaR* and *icaT* (≈60% and 66% of hits, respectively). We next wondered about the status of these genes in EIEC, which also harbors pINV. Using the PATRIC database, we identified EIEC strains 8-3-DC15 and 8-3-Ti3, which genomes were completely sequenced (22). Both strains displayed identical copies of *icaR* and *icaT* that mapped to loci homologous to those described above (Fig. S2). EIEC’s *icaR* and *icaT* were devoid of internal insertion or deletion and displayed 89% and 98% nucleotide identity, respectively, toward their M90T counterpart. Nonetheless, the MxiE box of *icaR* in EIEC carries a G6T mutation suggesting the activity of its promoter might be diminished (Fig. 1 and unpublished data). Notwithstanding this mutation, the status of *icaR* and *icaT* in EIEC appears similar to their M90T orthologs.

10.1128/msphere.00115-22.2 FIG S2 The locus of *icaR* and *icaT* are conserved. Loci of selected strains representative of *Shigella* subgroups and E. coli phylogroups for (A) *icaR* (pink). (B) *icaT* (red). Functional genes are in black. Insertion sequences (IS) are dark grey. Genes and pseudogenes encoding a hypothetical protein (hP) are burgundy. The *argW* (green) encodes the tRNA^Arg^ with the anticodon CCU. *shiE* (light grey) is a pseudogene. Download FIG S2, EPS file, 0.2 MB. Copyright © 2022 Silué and Campbell-Valois. 2022 Silué and Campbell-Valois. https://creativecommons.org/licenses/by/4.0/ This content is distributed under the terms of the Creative Commons Attribution 4.0 International license.

10.1128/msphere.00115-22.6 TABLE S1 Occurrence of *icaR* and *icaT* in selected strains of *Shigella* and Escherichia coli representative of the main phylogroups. Download Table S1, DOCX file, 0.02 MB. Copyright © 2022 Silué and Campbell-Valois. 2022 Silué and Campbell-Valois. https://creativecommons.org/licenses/by/4.0/ This content is distributed under the terms of the Creative Commons Attribution 4.0 International license.

10.1128/msphere.00115-22.7 TABLE S2 Global occurrence of *icaR* and *icaT* in *Shigella* subgroups. Download Table S2, DOCX file, 0.01 MB. Copyright © 2022 Silué and Campbell-Valois. 2022 Silué and Campbell-Valois. https://creativecommons.org/licenses/by/4.0/ This content is distributed under the terms of the Creative Commons Attribution 4.0 International license.

Furthermore, we observed the presence of *icaR* and *icaT* in all strains of phylogroups A, B1, and E that we scrutinized (23, 24). Likewise, three out of the four strains tested in phylogroup D harbored *icaR* and *icaT*, whereas IAI39 only harbored *icaT*. In contrast, five out of six strains belonging to phylogroup B2 were devoid of both genes, whereas the outlier strain O127:H6 E2348/69 only harbor *icaR* (Table S1). The *icaR*-negative B2 and D strains have in common a duplication of *argF* and a loss of *tabA*, *bdcR,* and *bdcA*, which set them apart from other E. coli scrutinized. Furthermore, the primary structure of IcaR and IcaT was rather well conserved as the pairwise sequence identities between E. coli homologs and *Shigella* M90T were 65 to 80% and 71 to 91%, respectively. In E. coli, IcaR orthologs were overall similar in length to YjgL from K-12 MG1655 (median of 475 residues versus 604 residues, respectively) (Fig. S3). The strains with the lowest molecular weight IcaR (e.g., W, UMN026, and E2348/69) displayed C-terminal truncations due to IS. The orthologs with higher molecular weight were due to internal and carboxy-terminal additions, which tended to be conserved among strains of the same phylogroups. On the other hand, the sequence conservation of IcaT was high and its length was homogenous across the full data set (mean ≈ 352) as well as upon comparing *Shigella* and E. coli sequences (means of 314 and 354, respectively), Sb227 being the single low-end outlier (Fig. S4). IcaT from all strains of phylogroup E and one strain of phylogroup D displayed a similar 71 residues carboxy-terminal truncation. A single internal addition of similar length and composition was observed at the end of the first half of the primary structure in all strains of phylogroups D and E. This suggests that some of the variations in the sequence of IcaR and IcaT were phylogroup specific whereas others, albeit less frequent, were shared between some phylogroups or were strain-specific. Taken together, these results indicated that *icaR* and *icaT* are prevalent in *E. coli*, although to a lesser extent in *Shigella*. In addition, *icaT* was more conserved than *icaR*.

10.1128/msphere.00115-22.3 FIG S3 The primary structure of IcaR is conserved in E. coli. Alignment of the primary structure of IcaR from strain representative of *Shigella* subgroups and E. coli phylogroups. The prefix of each sequence indicates the *Shigella* subgroups or the E. coli phylogroups to which each corresponding strain belongs. Apart from various carboxy-terminal truncations, the sequence is relatively well conserved. See the text for more details. Download FIG S3, EPS file, 0.7 MB. Copyright © 2022 Silué and Campbell-Valois. 2022 Silué and Campbell-Valois. https://creativecommons.org/licenses/by/4.0/ This content is distributed under the terms of the Creative Commons Attribution 4.0 International license.

10.1128/msphere.00115-22.4 FIG S4 The primary structure of IcaT is conserved in E. coli. Alignment of the primary structure of IcaT from strain representative of *Shigella* subgroups and E. coli phylogroups. The prefix of each sequence indicates the *Shigella* subgroups or the E. coli phylogroups to which each corresponding strain belongs. Apart from various carboxy-terminal truncations, the sequence is relatively well conserved. See the text for more details. Download FIG S4, EPS file, 0.6 MB. Copyright © 2022 Silué and Campbell-Valois. 2022 Silué and Campbell-Valois. https://creativecommons.org/licenses/by/4.0/ This content is distributed under the terms of the Creative Commons Attribution 4.0 International license.

### The introduction of *mxiE* and *ipgC* in E. coli activated the expression of *icaR* and *icaT*.

**FIG 4:**
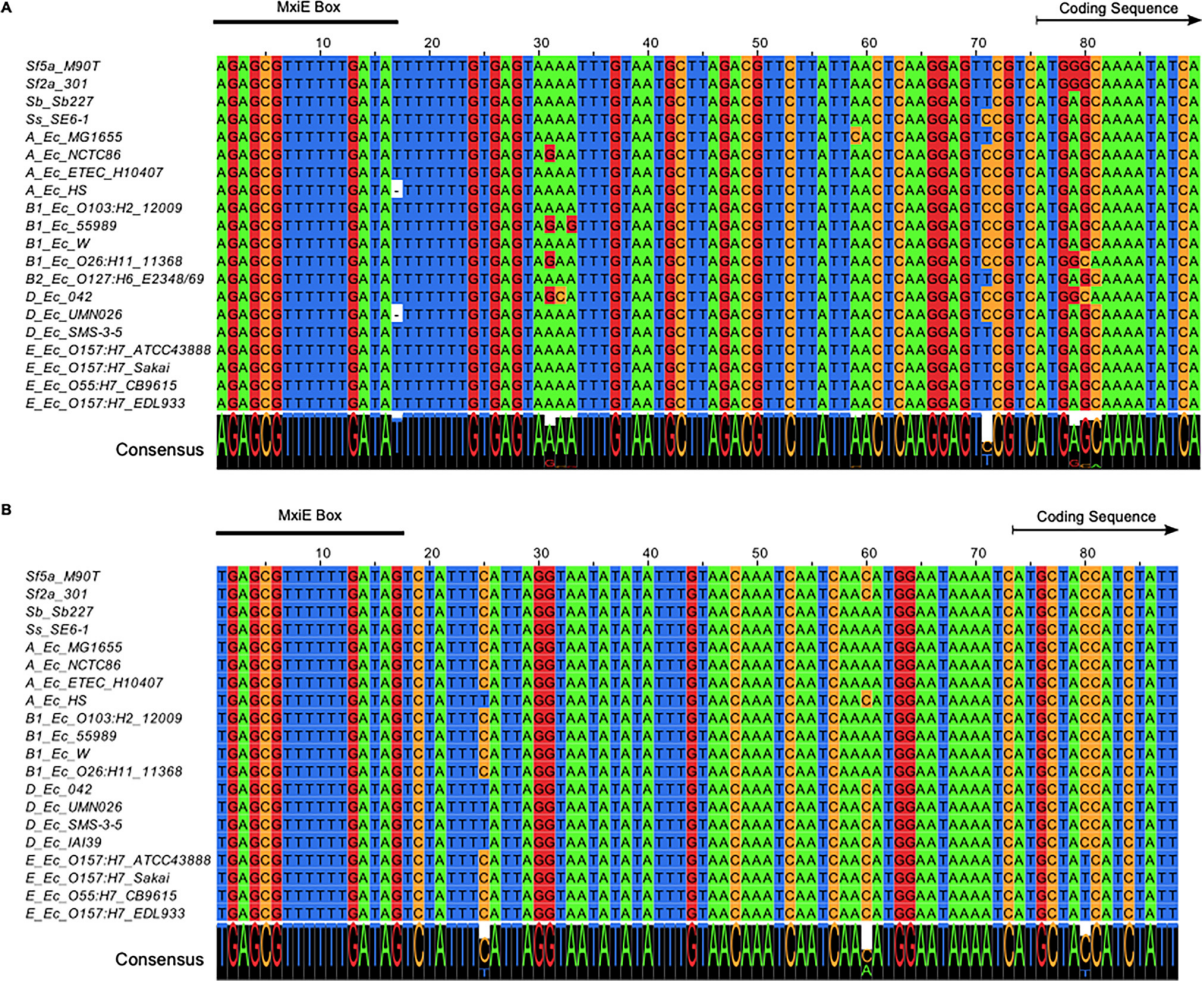
The MxiE box in *icaR* and *icaT* is well conserved. Alignment of the promoter, the 5’UTR, and the 5′ end of the coding sequence for (A) *icaR*. (B) *icaT*. The prefix of each sequence indicates the *Shigella* subgroups or the E. coli phylogroups to which each corresponding strain belongs. The region matching the MxiE box, and the beginning of the coding sequence are indicated. The consensus sequence represented in larger font at the bottom of each alignment indicated the strong conservation of the promoter and 5’ untranslated region of these genes.

**FIG 5:**
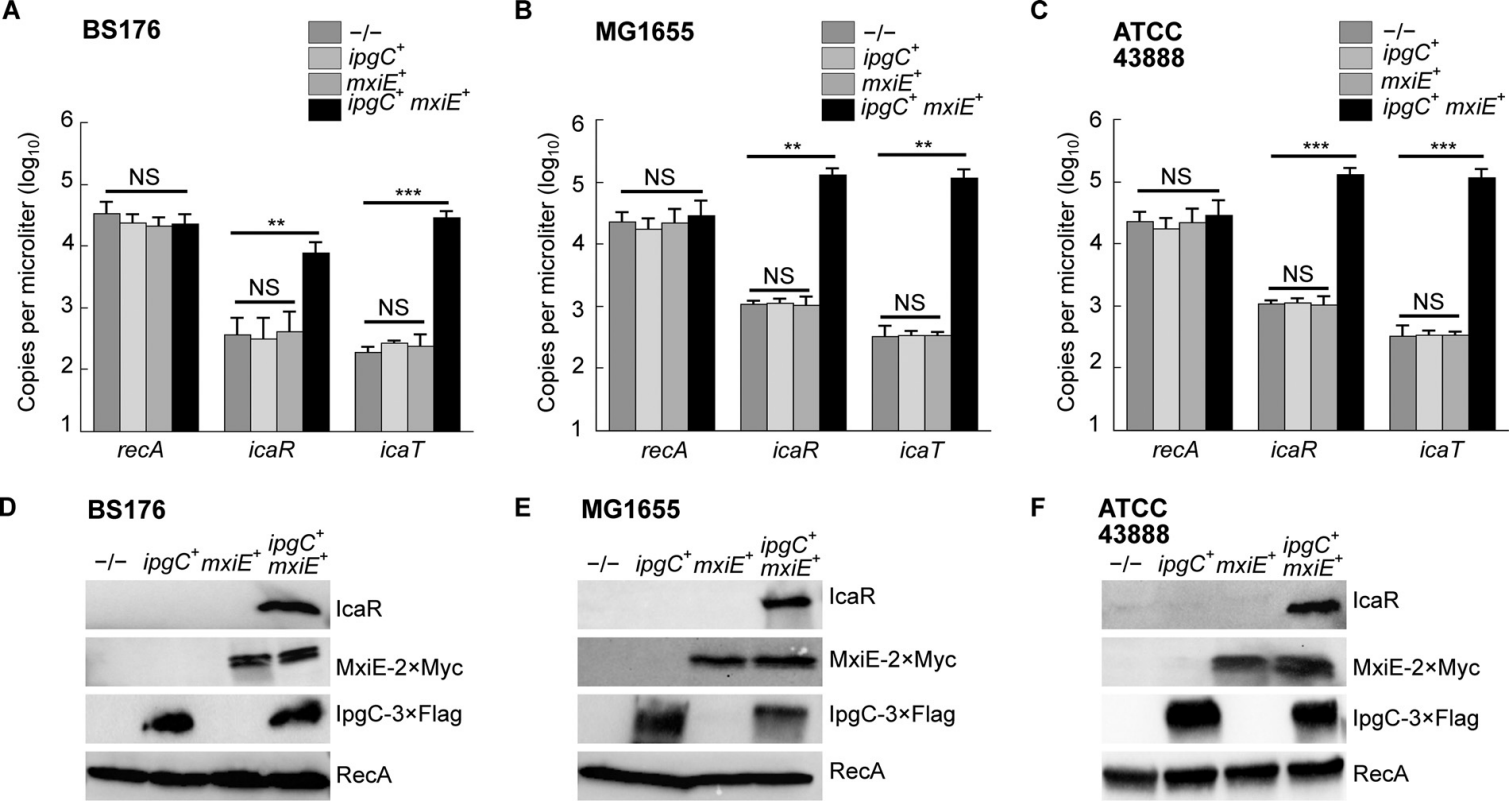
The coproduction of MxiE and IpgC activates the expression of *icaR* and *icaT* in E. coli. Quantification of the expression, as the number of copies of transcript per microliter, of *icaR*, *icaT,* and *recA* by ddPCR in −/−, *ipgC*^+^, *mxiE*^+^ or *ipgC*^+^ *mxiE*^+^ strains obtained by transformation with the relevant plasmids. (A) BS176, a plasmid cured derivative of S. flexneri 5a str. M90T. (B) E. coli K-12 str. MG1655, a representative of phylogroup A. (C) E. coli O157:H7 str. ATCC 43888, a representative of phylogroup E. The mean values and standard deviations based on three biological replicates are represented. The statistical significance of these data was tested with a one-way ANOVA and a Bonferroni correction for pairwise *post hoc* tests with a 95% confidence interval; *, *P* < 0.05; **, *P* < 0.01; ***, *P* < 0.001; NS, not significant. (D to F) Detection of the production of MxiE and IpgC and of IcaR from its endogenous locus by immunoblotting the TCL of strains described in (A to C). These results are representative of three independent experiments.

**FIG 6:**
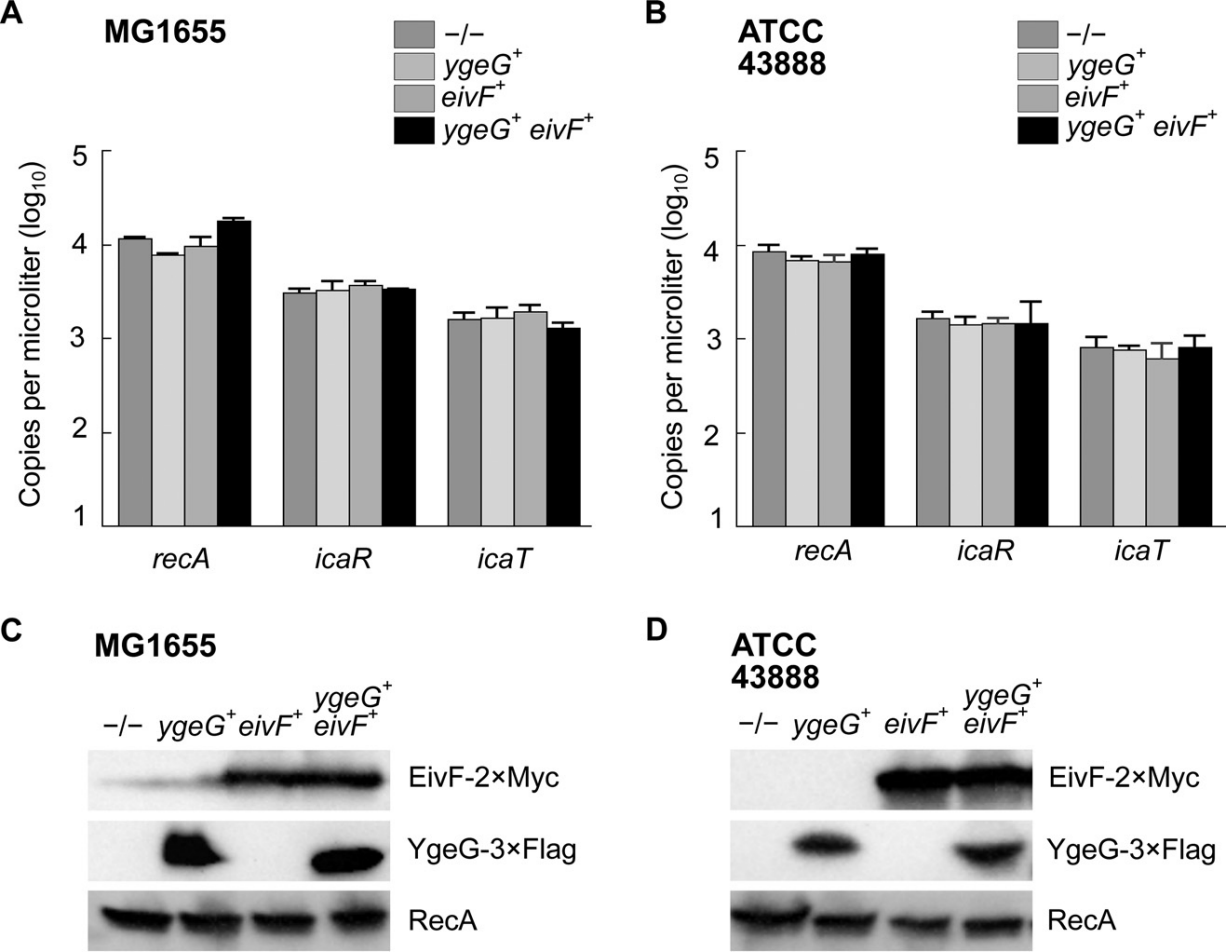
The coproduction of EivF and YgeG does not activate the expression of *icaR* and *icaT* in E. coli. Quantification of the expression, as the number of copies of transcript per microliter, of *icaR*, *icaT*, and *recA* by ddPCR in −/−, *ygeG*^+^, *eivF*^+^ or *ygeG*^+^ *eivF*^+^ strains obtained by transformation with the relevant plasmids. (A) E. coli K-12 str. MG1655. (B) E. coli O157:H7 str. ATCC 43888. The mean values and standard deviations based on three biological replicates are represented. The statistical significance of these data was tested with a one-way ANOVA and a Bonferroni correction for pairwise *post hoc* tests with a 95% confidence interval. There were no statistically significant variations in the data within any given group. (C to D) Detection of the production of YgeG and EivF by immunoblotting the TCL of strains described in (A and B). These results are representative of three independent experiments.

It is noteworthy that the 5′ UTR and the promoter region, notably the MxiE boxes identified in S. flexneri 5a str. M90T, were highly conserved in both *icaR* and *icaT* alignments, suggesting that these genes were functional in most non-*Shigella* E. coli (Fig. 4). Because E. coli does not harbor *mxiE* and *ipgC*, we reasoned that they should not express *icaR* and *icaT*. Therefore, we hypothesized that the ectopic production of MxiE and IpgC in E. coli should activate the expression of these genes. To test this, we selected a representative of phylogroup A, the commensal K-12 strain MG1655, a representative of phylogroup E, the O157:H7 strain ATCC 43888, and BS176, a virulence pINV-cured strain derived from M90T that does not produce MxiE and IpgC. Then, we introduced plasmids allowing the production of MxiE-2×Myc and IpgC-3×FLAG into these strains and measured the expression of *icaR* and *icaT* by ddPCR. These data indicated that *ipgC*^+^ *mxiE*^+^ cells expressed higher levels of *icaR* and *icaT* than the control strains, whereas the expression of the housekeeping gene *recA* was unaffected (Fig. 5A to C). Using the polyclonal antibody introduced earlier, we confirmed the increased production of IcaR from its endogenous locus in *ipgC*^+^ *mxiE*^+^ cells (Fig. 5D to F). In both E. coli strains, the apparent MW of the protein encoded by *icaR* was approximately 70 kDa, as expected due to the integrity of their coding sequences, which contrast with their truncated *Shigella* counterpart (Fig. S3 and S5). The E. coli T3SS 2 (ETT2) was present in part or whole in the chromosome of most E. coli phylogroups. The ETT2 harbored distant homologs of *mxiE* and *ipgC* named *eivF* and *ygeG*, respectively. Thus, we wondered whether they might also be endowed with the capacity to activate *icaR* and *icaT*. To test this hypothesis, we introduced *eivF* and *ygeG* in MG1655 and ATCC 43888 (Fig. 6). The data indicated that the coproduction of EivF and YgeG did not activate the expression of *icaR* and *icaT*, thereby highlighting the unique capacity of MxiE and IpgC to do so.

10.1128/msphere.00115-22.5 FIG S5 Full membrane of immunoblotting corresponding to Fig. 5D to F. Detection of IcaR produced from its endogenous locus by immunoblotting the TCL of strains described in Fig. 5. (A) BS176, full membrane corresponding to Fig. 5D. (B) MG1655, full membrane corresponding to Fig. 5E. (C) ATCC 43888, full membrane corresponding to Fig. 5F. Download FIG S5, EPS file, 1.4 MB. Copyright © 2022 Silué and Campbell-Valois. 2022 Silué and Campbell-Valois. https://creativecommons.org/licenses/by/4.0/ This content is distributed under the terms of the Creative Commons Attribution 4.0 International license.

## DISCUSSION

The data presented here suggest that *icaR* and *icaT* are regulated by MxiE and IpgC in *Shigella*. Both genes possess a functional MxiE box that is essential for their expression. Furthermore, the corresponding proteins IcaR and IcaT are secreted by the T3SA in a chaperone-independent manner similar to other late substrates B (5). As expected of T3SA-mediated secretion, the secretion signal of IcaR and IcaT is located at their N terminus. Therefore, the chromosome genes *icaR* and *icaT* encode T3SA substrates that were previously unknown. Before this work, a subset of IpaH genes were the only known cases of chromosome-encoded T3SA substrates in *Shigella* (5). Chromosomal *ipaH* are thought to have resulted from the duplication of pINV *ipaH* genes with which they share striking similarities. Because they do not have homologs on the plasmid, the origin of *icaR* and *icaT* is difficult to pinpoint and, hence, deserving of a separate discussion in another paragraph. Nevertheless, it is noteworthy that the 3′ end of the *icaR* open reading frame is truncated by an IS sequence in *Shigella*, suggesting that this happened after they diverged from other phylogroups. Because E. coli strains are devoid of chromosomal *ipaH*, we reasoned that the acquisition of *icaR* and *icaT* preceded the transfer of *ipaH* genes to the chromosome of *Shigella*. Generally, *icaR* and *icaT* are frequently disrupted by the landing of additional IS in S. boydii, S. dysenteriae, and S. sonnei. In S. flexneri, both genes are more frequently integral, notwithstanding the 3′ deletion of *icaR* discussed earlier. The resulting truncated protein, however, is sufficiently stable to be detected by immunoblotting, suggesting it might still be partly functional. In brief, *icaR* and *icaT* are expressed to low levels (5, 13), and conserved mostly in S. flexneri, which indicates they are not under strong evolutionary pressure in *Shigella*. Hence, a role for IcaR or IcaT in the pathogenesis of S. flexneri cannot be discarded, but it is likely minor.

Furthermore, *icaR* and *icaT* are present in all the tested E. coli strains that belong to phylogroups A, B1, and E. Our data suggest that *icaR* and *icaT* can be activated by the ectopic expression of *mxiE* and *ipgC* in a strain of phylogroup A or E. We expect this phenomenon to be widespread because *icaR* and *icaT* orthologs are associated with a conserved MxiE box irrespective of their phylogroups. In contrast, most strains of phylogroup B2 harbor neither *icaR* nor *icaT*. Several independent phylogenetic studies indicated that B2 is the most ancient phylogroup of E. coli (23–27). Instead, phylogroups A, B1, and E constitute a more recent radiation. The presence of *icaR* and *icaT* in these three phylogroups coupled with their absence in the B2 agrees with this model of the evolution of E. coli (27). The strain O127:H6 E2348/69 in phylogroup B2, however, harbor *icaR*, suggesting the picture is more complex. Furthermore, *icaT* is present in the four D strains scrutinized, whereas *icaR* is present in three of them. This discrepancy might stem from the genetic diversity of this phylogroup considered to be the closest to the primordial E. coli (25–27). It is also noteworthy that the *icaR*-negative B2 and D strains have in common a remodeled *argF* locus. Therefore, an alternative model is that *icaR* was present in the E. coli lineage before the radiation of the B2 phylogroup but was later lost in most B2 strains and some D strains through shuffling of the *argF* locus. In contrast, the specific absence of *icaT* in the B2 phylogroup coupled with the integrity of its *fadL* locus suggests that *icaT* appeared in the last common ancestor of the other phylogroups after B2 had branched off. Taken together, these data suggest that *icaR* and then, *icaT* were inserted in the chromosome through two distinct horizontal transfer events.

Comparative phylogeny of pINV genes and chromosomal genes suggested that the ancestor of phylogroup A and B1 harbored an ancestor of pINV (28). This makes pINV a potential source of T3SS associated genes in E. coli. An alternative source is the ETT2, which is ubiquitous in E. coli, except in the B2 phylogroup (29). Whereas the ETT2 locus is moderately or severely disrupted within phylogroups A and B1, it is often integral in the phylogroups D and E, but evidence supporting its secretion activity is lacking (30, 31). Besides, the GC content of *icaR* and *icaT* (35.7%, 34.9%, respectively) is lower than that of the chromosome (50.9%), whereas it is comparable with those of the entry region of pINV (34.2%) and the ETT2 (36.9%) (30). Indeed, the expression of *icaR* and *icaT* is controlled by a MxiE box whose occurrence and that of the corresponding transcription activator MxiE and its co-activator IpgC has been solely associated with the entry region of pINV and of the related pEM148 (6, 32). The ETT2 harbors distant homologs to MxiE and IpgC named EivF and YgeG, highlighting the possibility that they might also regulate *icaR* and *icaT*. In contrast with this hypothesis, we found the coproduction of EivF and YgeG did not activate the expression of *icaR* and *icaT*. Furthermore, our data indicate that IcaR and IcaT are substrates of the T3SA encoded by pINV. This argument does not favor pINV over ETT2 as the origin of these genes, because substrates of one type of T3SA were shown to be substrates for others (33, 34). The protein encoded by *icaT* has low homology with transposases associated with IS (35). Nonetheless, the top-scoring match of IcaT in the IS finder is not toward the transposase of an IS family present in pINV, but rather toward a clostridia transposase of the IS256 family (36). In eukaryotes, transposases distantly related to those found in IS are implicated in gene fusion events that contributed to the emergence of new biological functions (37). Transposases also play a role in the evolution of new gene functions in prokaryotes, as suggested for CRISPR/Cas (38). Similarly, *icaT* might have resulted from the capture of a transposase domain by the T3SS. On the other hand, PFAM domains search with full-length IcaR (YjgL) from K-12 MG1655 revealed a statistically insignificant match with a short stretch of the central domain of the Salmonella T3SS effector SopA. This region is, however, absent of *Shigella* IcaR due to the carboxy-terminal truncation described above. Considering these observations, we cannot discard that *icaR* and *icaT* originated from neither pINV nor ETT2, but rather from an unknown mobile element. Given the paucity of functional MxiE boxes in the chromosome (12), we reasoned that whatever may be the origin of *icaR* and *icaT*, they nonetheless coevolved to some extent with the T3SS encoded in pINV or a relative, which eventually favored the installation of a MxiE box in their promoter. The conservation of *icaR* and *icaT* in E. coli is intriguing. Indeed, the absence of *mxiE* and *ipgC* silenced these genes in E. coli devoid of pINV. One could argue that precisely due to this silencing, these genes are under neutral selection pressure in E. coli. In contrast, in *Shigella* their cognate proteins could potentially interfere with virulence by competing with other substrates for T3SA-mediated secretion, thus introducing a negative selection pressure that might have contributed to the disruption of *icaR* and *icaT* in this pathovar. Taken together, these observations bring additional evidence that *icaR* and *icaT* did not acquire functions worthy of conservation.

In summary, *icaR* and *icaT* are ancient T3SS-associated genes that were already present in the chromosome of the last common ancestor of *Shigella* and several E. coli strains belonging mainly to phylogroups A, B1, D, and E. *icaR* and *icaT* are expressed to low levels in *Shigella* and only when T3SA are active, explaining why they had escaped attention thus far. Because IcaR and IcaT are T3SA substrates, they may act as effectors within host cells, although a function in the bacterial cytosol cannot be ruled out. In all cases, their potential role in *Shigella* would be restrained by their limited conservation and by their low production. Alternatively, the properties of *icaR* and *icaT* might suggest they are fossil genes or on the path to becoming so.

## MATERIALS AND METHODS

### Bacterial strains.

Shigella flexneri str. M90T WT and its isogenic mutants *ipaB4*, *ipaB4* Δ*mxiE, ipaB4* Δ*ipgC*, Δ*ipaD*, Δ*mxiD*, or the plasmid cured strain BS176 were obtained from Philippe Sansonetti and Claude Parsot (16, 39–42). Shigella flexneri 2a str. 2457T and its isogenic mutants Δ*ipgA*, Δ*ipgE*, Δ*spa15*, and Δ*ipgA* Δ*ipgE* Δ*spa15* (ΔΔΔ) were obtained from Cammie F. Lesser (8). E. coli K-12 str. MG1655 (DSM18039) and O157:H7 (ATCC 43888) were obtained from the DSMZ and ATCC, respectively. *Shigella* strains and E. coli O157:H7 strain ATCC 43888 were routinely grown on tryptic soy agar (TSA) and tryptic soy broth (TSB) with or without antibiotics. All other E. coli strains were grown on Luria-Bertani agar or broth supplemented with the appropriate antibiotics when required.

### Plasmids.

Plasmids used in this study are described in Table S3. Briefly, the promoter and the coding region of *icaT* and *icaR* were obtained by PCR with primer pairs HMIO117/HMIO118 and HMIO119/HMIO120 (Table S4), respectively, using a small volume of liquid culture of S. flexneri 5a str. M90T. The resulting amplicons were digested by KpnI and Kpn2I and inserted by ligation into pUC18Δ-3×Flag (13). Several derivatives of these constructs were made using mutagenesis PCR, Gibson assembly (NEBuilder HiFi, New England Biolab), or both (Table S4): (i) three punctual mutations at conserved positions of the MxiE box (G6C, T12A, and A16C) were independently introduced (11); (ii) the endogenous promoters were replaced with the *lacZ* promoter (*lacZp*); (iii) the 5′ end of the coding sequence downstream of the start codon was deleted to generate amino-terminal truncations of 5, 10 and 20 residues; (iv) using NEBuilder, *icaR* and *icaT* were inserted at the 5′ end of the coding sequence of bla_TEM3_ M182T devoid of the region encoding its signal peptide (20). The 3’end of the coding sequence (upstream of bp 63) of *icaR* and *icaT* was then deleted by mutagenesis PCR. The coding sequence of IpgC was amplified by PCR from a colony of S. flexneri str. M90T and cloned by ligation following restriction digest with BglII and BamHI into pUC18.1, a derivative of pUC18; the 3′ end 3×FLAG was then inserted by mutagenesis PCR (FXCV unpublished work). MxiE-Myc was subcloned by restriction digest with EcoRI and XbaI into pSU2.1 a derivative of pSU2718 (FXCV unpublished data). EivF and YgeG were amplified from O157:H7 str. ATCC 43888 and cloned by Gibson assembly in place of *mxiE* and *ipgC*, respectively. All constructs were verified by Sanger sequencing (Génome Québec).

10.1128/msphere.00115-22.8 TABLE S3 The plasmids used in this work. Download Table S3, DOCX file, 0.02 MB. Copyright © 2022 Silué and Campbell-Valois. 2022 Silué and Campbell-Valois. https://creativecommons.org/licenses/by/4.0/ This content is distributed under the terms of the Creative Commons Attribution 4.0 International license.

10.1128/msphere.00115-22.9 TABLE S4 The oligonucleotides used in this work. Download Table S4, DOCX file, 0.02 MB. Copyright © 2022 Silué and Campbell-Valois. 2022 Silué and Campbell-Valois. https://creativecommons.org/licenses/by/4.0/ This content is distributed under the terms of the Creative Commons Attribution 4.0 International license.

### Detection of the expression of IcaR and IcaT by immunoblotting.

M90T WT, *ipaB4*, *ipaB4* Δ*ipgC*, and *ipaB4* Δ*mxiE* isogenic strains harboring either one of plasmids pNS1-8 (Table S3) were inoculated in TSB supplemented with ampicillin (100 μg/mL) and incubated overnight at 37°C with shaking at 250 rpm. Next, the resulting cultures were diluted 1:1 in laemmli 2× and heated at 95°C for 5 min and ran into 4 to 15% polyacrylamide gradient SDS-PAGE gels (Bio-Rad, number 456-8086). Immunoblotting was performed as described in (13), using the following antibodies as indicated: primary antibodies: 1/5000 mouse anti-FLAG (Sigma. number F3165), 1/10000 rabbit anti-IpaH, and 1/1000 mouse anti-RecA (MBL, number ARM191); secondary antibodies: 1/25000 (except for RecA 1/10000) anti-Mouse IgG-HRP (Jackson Immunoresearch, number 115-035-003), and 1/10000 anti-Rabbit IgG-HRP (Jackson Immunoresearch, number 111-035-003).

### Immunoblotting assay to measure constitutive secretion of IcaR and IcaT.

The secretion assay was performed as previously described with slight modifications indicated below (16). M90T WT, Δ*mxiD*, and Δ*ipaD* isogenic strains harboring plasmids pNS9 or pNS10 were inoculated in TSB supplemented with ampicillin (100 μg/mL) and incubated overnight at 30°C with shaking at 250 rpm. The next morning, these outgrowths were subcultured 1:100 into 4 mL of TSB and incubated for 4 h at 37°C. The optical density at 600 nm (OD_600_) was used to normalize the volume of each culture from which the TCL, as described above, and the secreted fraction (SF) were prepared. To isolate the SF, 2 mL of subcultures was centrifuged for 10 min at 17000 × *g*. The supernatant was transferred to a fresh microcentrifuge tube and the centrifugation was repeated a second time to remove residual cells. Then, 1.4 mL of the resulting supernatant was incubated overnight at 4°C in 10% (vol/vol) trichloroacetic acid (TCA) and centrifuged for 10 min at 14000 × *g*. The resulting protein pellet was washed twice with 200 μL of cold acetone (–20°C) and centrifuged for 5 min at 14000 × *g*. The pellet was air-dried for 5 min using a dry bath set to 95°C to evaporate acetone traces. The pellets were resuspended in 50 μL 1× laemmli buffer, loaded on an SDS-PAGE, and immunoblotted as described above. The membranes were blotted with anti-FLAG and anti-RecA antibodies described above, and with 1/1000 mouse anti-IpaC (clone N9) (43). This antibody was obtained from Armelle Phalipon. The secondary antibody anti-mouse IgG-HRP was used as described above.

### Immunoblotting assay to measure Congo red-induced secretion of IcaR.

The secretion assay was performed as previously described with the slight modifications indicated below (17). Briefly, Μ90Τ WT and Δ*mxiD* harboring plasmids pNS9, 11, 13, or 15 were incubated overnight at 30°C and subcultured at 37°C, as described for the constitutive secretion assay. Using OD600, an equivalent number of cells from each culture was pelleted, resuspended in 2 mL of 25 μM Congo red (CR) diluted in phosphate-buffered saline (PBS), and incubated for 30 min at 37°C. The TCL, SF, SDS-PAGE, and immunoblotting were performed as described above. The only notable change was that 1.8 mL of the supernatant of each culture was precipitated with the TCA to isolate the SF.

### Immunoblotting assay to measure Congo red-induced secretion of IcaT.

Μ90Τ WT and Δ*mxiD* harboring plasmids pNS10, 12, 14, or 16 were incubated overnight as described in the previous section. The resulting outgrowths were subcultured 1/100 in 100 mL TSB and incubated for 2 h at 37°C. Then, the culture of each strain was split into two groups of 50 mL each. In the first group, CR was adjusted to 10 μM to induce the secretion, whereas in the second group, serving as a negative-control, no CR was added. Both groups were then incubated at 37°C for 4h. The resulting cultures were centrifuged for 20 min at 4500 × *g*. Forty milliliters of the resulting supernatant was used to obtain the SF using TCA precipitation, as described above. The protein pellets were resuspended in 400 μL of 1× laemmli and 4 μL of NaOH (8 M) to neutralize the pH. Samples were analyzed by immunoblotting as described above.

### β-lactamase secretion assay.

Δ*mxiD* and Δ*ipaD* were transformed with pNS17, pNS19, or pSU2.1tt ospD1sh M31L bla_TEM3_ M182T. The latter is described in reference (20) and served as a control. The nitrocefin β-lactamase secretion assay was performed as previously described (7, 20). The absorbance of nitrocefin hydrolysis was measured at 486 nm into a 96-well microplate (Greiner, number 655096) with a Synergy H4 plate reader (Agilent).

### Activation of *icaR* and *icaT* in E. coli.

The M90T derivative plasmid-cured strain BS176, E. coli K-12 str. MG1655 and O157:H7 str. ATCC 43888 harboring plasmids pNS21 (*ipgC*^+^), pNS22 (*mxiE*^+^), both (*ipgC*^+^ *mxiE*+), or none (−/−) or pNS23 (*ygeG*^+^), pNS24 (*eivF*^+^), both (*ygeG*^+^ *eivF*^+^), or none (−/−) were cultivated, as described in the previous section, except that pNS22 and pNS24 were maintained with 30 μg/mL chloramphenicol. Primer design, RNA extraction, cDNA synthesis, and ddPCR were performed as previously described (13). We used the primers ddO1/ddO2, ddO7/ddO8, and qPCR87/qPCR88 to amplify *icaT*, *icaR,* and *recA*, respectively. Care was taken to select primers annealing to regions that were identical across the strains analyzed. To perform the ddPCR, template cDNA of BS176, ATCC 43888, MG1655 were diluted 1/75, 1/100, and 1/250, respectively. The optimal annealing temperature of 58°C was determined through a temperature gradient experiment. The culture used for preparing the cDNA was also used for the detection of IcaR by immunoblotting. The TCL was prepared as described above, but 1 mL of BS176 and ATCC 43888 cultures were resuspended in 100 μL of laemmli (10× concentrate). Primary antibodies used: 1/500 rabbit polyclonal anti-IcaR antibody raised against residues 143 to 158: SDGSTNRYEGKSFERK (MÉDIMABS, Montréal, QC; custom made antibody, number pAb170-Anti P2); 1/20000 mouse anti-Myc (Genescript, number A00704); other primary and secondary antibodies are described above.

### Bioinformatics.

Strains representative of each *Shigella* subgroup and E. coli phylogroup with available whole-genome sequencing were mined for *icaR* and *icaT* using protein and nucleotide BLAST searches (44), and S. flexneri 5a str. M90T *icaR* and *icaT* as queries. Nucleotide BLAST (BLASTn) searches were also performed by excluding *Shigella* and E. coli to identify other species that harbored the genes. These were complemented with BLASTn against species related to E. coli such as Escherichia marmotae, Escherichia albertii, Escherichia fergusonii, or Salmonella spp. The loci of selected E. coli strains were obtained through the NCBI Sequence Viewer (45) and represented using Snapgene (Insightful Science). Protein and nucleotide alignments were done with MUSCLE in Jalview using ClustalX coloring (46, 47). The region spanning from the MxiE box to the end of the coding sequence of *icaR* and *icaT* from str. M90T was used as a query in successive nucleotide BLAST searches within each *Shigella* subgroup. We categorized *icaR* and *icaT* in each strain identified in our list as integral, moderately, or highly disrupted. The thresholds used to establish those categories were as follows. Integral: number of hits with greater than or equal to 99% query coverage, greater than or equal to 98% sequence identity, and gap intolerance. Moderately disrupted: number of hits with greater than or equal to 80% query coverage subtracted by integral genes. Highly disrupted: total hits subtracted by integral and moderately disrupted genes. The percentage in each category was computed using the number of total hits as the denominator.
