# Supplementary material for: icaR and icaT are Ancient Chromosome Genes Encoding Substrates of the Type III Secretion Apparatus in Shigella flexneri
Source: mSphere. 2022 May 2;7(3):e00115-22. doi: 10.1128/msphere.00115-22 (PMC9241512; doi:10.1128/msphere.00115-22)
Supplement: TABLE S1 [file msphere.00115-22-s0001.docx]

**Table S1.** Occurrence of *icaR* and *icaT i*n Shigella and various *Escherichia coli* strains belonging to the main phylogroups.

| **Phylogroups** | **Strains name** | **Accession number** | ***icaR*** | ***icaT*** |
| --- | --- | --- | --- | --- |
| **Shigella** | S. flexneri 5a str. M90T | NZ_CM001474.1 | Yes | Yes |
|  | S. flexneri 2a str. 301 | NC_004337.2 | Yes | Yes^1^ |
|  | S. boydii Sb227 | NC_007613.1 | Yes | Yes |
|  | S. sonnei strain SE6-1 | NZ_CP055292.1 | Yes | Yes |
|  | S. sonnei Ss046 | CP000038.1 | Yes | Pseudo.^2^ |
|  | S. dysenteriae Sd197 | NC_007606.1 | No^3^ | Pseudo.^4^ |
| **A** | *E. coli* str. K-12 substr. MG1655 | U00096.3 | Yes | Yes |
|  | *E. coli* strain NCTC86 | NZ_CP019778.1 | Yes | Yes |
|  | *E. coli* ETEC H10407 | NC_017633.1 | Yes | Yes |
|  | *E. coli* HS | NC_009800.1 | Yes | Yes |
| **B1** | *E. coli* O103:H2 str. 12009 | NC_013353.1 | Yes | Yes |
|  | *E. coli* 55989 | NC_011748.1 | Yes | Yes |
|  | *E. coli* W | NC_017635.1 | Yes | Yes |
|  | *E. coli* O26: H11 str. 11368 | NC_013361.1 | Yes | Yes |
| **B2** | *E. coli* O127:H6 str. E2348/69 | NC_011601.1 | Yes^5^ | No |
|  | *E. coli* 536 | NC_008253.1 | No | No |
|  | *E. coli* SE15 | NC_013654.1 | No | No |
|  | *E. coli* ABU 83972 | NC_017631.1 | No | No |
|  | *E. coli* CFT073 | NZ_CP051263.1 | No | No |
| **D** | *E. coli* 042 | NC_017626.1 | Yes | Yes |
|  | *E. coli* UMN026 | NC_011751.1 | Yes | Yes |
|  | *E. coli* SMS-3-5 | NC_010498.1 | Yes | Yes |
|  | *E. coli* IAI39 | NC_011750.1 | No | Yes |
| **E** | *E. coli* O157:H7 strain ATCC43888 | NZ_CP041623.1 | Yes | Yes |
|  | *E. coli* O157:H7 str. Sakai | NC_002695.1 | Yes | Yes |
|  | *E. coli* O55:H7 str. CB9615 | NC_013941.1 | Yes | Yes |
|  | *E. coli* O157:H7 str. EDL933 | NZ_CP008957.1 | Yes | Yes |

^1^ An IS sequence is inserted between the MxiE box-containing promoter and the coding sequence, which is integral. *icaT* if still functional may be co-transcribed with the IS.

^2^ An IS is between the MxiE box and the coding sequence; the coding sequence is disrupted by 27 stops codons.

^3^ Three IS are located in the locus. The coding sequence is almost completely removed, but the MxiE box is still present upstream of one of the IS.

^4^ IS induced a large truncation at the 5’ end of the coding sequence; the MxiE box is absent.

^5^ This strain possesses the largest truncation at the 3’ end of *icaR*.
