# Supplementary material for: icaR and icaT are Ancient Chromosome Genes Encoding Substrates of the Type III Secretion Apparatus in Shigella flexneri
Source: mSphere. 2022 May 2;7(3):e00115-22. doi: 10.1128/msphere.00115-22 (PMC9241512; doi:10.1128/msphere.00115-22)
Supplement: TABLE S2 [file msphere.00115-22-s0002.docx]

**Table S2.** Occurrence of *icaR* and *icaT* in Shigella subgroups^1^.

| ***icaR*** | | | | | |
| --- | --- | --- | --- | --- | --- |
|  |  | **Total hits**  **(counts)** | **Integral (%)^2^** | **Moderate disruption (%)^3^** | **High**  **disruption (%)^4^** |
|  | **boydii** | 26 | 3.8 | 96.2 | 0 |
|  | **dysenteriae** | 23 | 0 | 60.9 | 39.1 |
|  | **flexneri** | 56 | 60.7 | 35.7 | 3.6 |
|  | **sonnei** | 43 | 0 | 100 | 0 |
| ***icaT*** | | | | | |
|  | **boydii** | 27 | 11.1 | 81.5 | 7.4 |
|  | **dysenteriae** | 21 | 0 | 23.8 | 76.2 |
|  | **flexneri** | 63 | 66.7 | 28.6 | 4.8 |
|  | **sonnei** | 43 | 2.3 | 97.7 | 0 |

^1^ *icaR* and *icaT* from M90T and spanning from the MxiE box to the stop codon were used as queries.

^2^ Integral genes were defined as hits ≥99% query coverage, ≥98% pairwise sequence identity, and no gap; Integral genes (counts)/total hits (counts)*100= percentage

^3^ Genes with moderate disruption were defined as hits ≥80% query coverage subtracted by the integral genes; Moderately disrupted genes (counts)/total hits (counts)*100= percentage.

^4^ Genes with high disruption were defined as hits <80% query coverage; highly disrupted genes (counts)/total hits (counts)*100= percentage.
