# Supplementary material for: icaR and icaT are Ancient Chromosome Genes Encoding Substrates of the Type III Secretion Apparatus in Shigella flexneri
Source: mSphere. 2022 May 2;7(3):e00115-22. doi: 10.1128/msphere.00115-22 (PMC9241512; doi:10.1128/msphere.00115-22)
Supplement: TABLE S3 [file msphere.00115-22-s0003.docx]

**Table S3.** The plasmids used in this work.

| **Plasmid ID** | **Plasmid name** | **Properties** | **Reference** |
| --- | --- | --- | --- |
| pNS1 | pUC18Δ *icaR*-3xFlag | *icaR*p, str. M90T | This study |
| pNS2 | pUC18Δ *icaT*-3xFlag | *icaT*p, str. M90T | This study |
| pNS3 | pUC18Δ G6C *icaR*-3xFlag | G6C MxiE-Box mutation | This study |
| pNS4 | pUC18Δ G6C *icaT*-3xFlag | G6C MxiE-Box mutation | This study |
| pNS5 | pUC18Δ T12A *icaR*-3xFlag | T12A MxiE-Box mutation | This study |
| pNS6 | pUC18Δ T12A *icaT*-3xFlag | T12A MxiE-Box mutation | This study |
| pNS7 | pUC18Δ A16C *icaR*-3xFlag | A16C MxiE-Box mutation | This study |
| pNS8 | pUC18Δ A16C *icaT*-3xFlag | A16C MxiE-Box mutation | This study |
| pNS9 | pUC18Δ *lacZ*p::*icaR*-3xFlag | *lacZ*p fusion with *icaR*’s CDS | This study |
| pNS10 | pUC18Δ *lacZ*p::*icaT*- 3xFlag | *lacZ*p fusion with *icaT*’s CDS | This study |
| pNS11 | pUC18Δ *lacZ*p::*icaR* Δ15-3xFlag | Residues 2-6 (D5) of IcaR are truncated | This study |
| pNS12 | pUC18Δ *lacZ*p::*icaT* D15-3xFlag | Residues 2-6 (D5) of IcaR are truncated | This study |
| pNS13 | pUC18Δ *lacZ*p::*icaR* D30-3xFlag | Residues 2-11 (D10) of IcaR are truncated | This study |
| pNS14 | pUC18Δ *lacZ*p::*icaT* D30-3xFlag | Residues 2-11 (D10) of IcaT are truncated | This study |
| pNS15 | pUC18Δ *lacZ*p::*icaR* D60-3xFlag | Residues 2-21 (D20) of IcaR are truncated | This study |
| pNS16 | pUC18Δ *lacZ*p::*icaT* D60-3xFlag | Residues 2-21 (D20) of IcaT are truncated | This study |
| pNS17 | pSU2.1tt *icaR*1-63-bla_TEM3_ M182T | *icaR*p; produces IcaR residues 1-21 fused to bla | This study |
| pNS19 | pSU2.1tt *icaT*1-63- bla_TEM3_ M182T | *icaT*p; produces IcaT residues 1-21 fused to bla | This study |
| **Plasmid ID** | **Plasmid name** | **Properties** | **Reference** |
| N/A | pSU2.1tt *ospD1*sh M31L bla_TEM3_ M182T | *ospD1*p; produces OpsD1 residues 1-80 M31L fused to bla | (20) |
| pNS21 | pUC18.1 *lacZ*p::*ipgC*-3xFlag | str. M90T | Unpublished work by FXCV |
| pNS22 | pSU2.1 *lacZ*p::*mxiE*-2xMyc | str. M90T | Unpublished work by FXCV |
| pNS23 | pUC18.1 *lacZ*p::*ygeG*-3xFlag | O157:H7 str. ATCC43888 | This study |
| pNS24 | pSU2.1 *lacZ*p::*eivF-*2xMyc | O157:H7 str. ATCC43888 | This study |
