# Supplementary material for: icaR and icaT are Ancient Chromosome Genes Encoding Substrates of the Type III Secretion Apparatus in Shigella flexneri
Source: mSphere. 2022 May 2;7(3):e00115-22. doi: 10.1128/msphere.00115-22 (PMC9241512; doi:10.1128/msphere.00115-22)
Supplement: TABLE S4 [file msphere.00115-22-s0004.docx]

**Table S4.** The oligonucleotides used in this work.

| **Primer** | **Sequence (5’-3’)** | **Description** |
| --- | --- | --- |
| HMIO117  HMIO118 | AGAGAGGGTACCTAACGCGTTCGCCTGGATAAAG  AGAGAGTCCGGAAATATCATCAAAATTAACACCTAGTGCAG | Amplification of *icaT* from M90T |
| HMIO119  HMIO120 | AGAGAGGGTACCTGACTTTTCGCCGTAAATAACTCC  AGAGAGTCCGGACCCTTTATTCAATGCTTTTTCTAATAA ATCC | Amplification of *icaR* from M90T |
| HMIO121  HMIO122 | AAGTGTAAAGCCTGGGGTGCCTAAGGTACCGAGCTC GAATTCAAA  TATGCTTCCGGCTCGTATGTTGTGTGGAAACAAATCAAT CAACATGGAATAAAATC | Insertion of lac-promoter upstream of *icaT* |
| HMIO121  HMIO123 | AAGTGTAAAGCCTGGGGTGCCTAAGGTACCGAGCTC GAATTCAAA  TATGCTTCCGGCTCGTATGTTGTGTGGAACGTT CTTATTAACTCAAGGAGTTCGT | Insertion of lac-promoter upstream of *icaR* |
| HMIO129  HMIO130 | AGATAGTCTATTTCATTAGGTAATATATATTTG  AAAAACGCTCATTTATTTAGA | *icaT* MxiE box mutation T12A |
| HMIO131  HMIO132 | AGATATTTTTTTGTGAGTAAAATTTG  AAAAACGCTCTTTTCACA | *icaR* MxiE box mutation T12A |
| HMIO133  HMIO134 | CTTTTTTGATAGTCTATTTCATTAGG  GCTCATTTATTTAGAACCTATC | *icaT* MxiE box mutation G6C |
| HMIO136  HMIO137 | CTTTTTTGATATTTTTTTGTGAG  GCTCTTTTCACAAACTCG | *icaR* MxiE box mutation G6C |
| HMIO139  HMIO141 | CGTCTATTTCATTAGGTAATATATATTTGT  ATCAAAAAACGCTCATTTATTTAG | *icaT* MxiE box mutation A16C |
| HMIO142  HMIO144 | CTTTTTTTGTGAGTAAAATTTGTAA  ATCAAAAAACGCTCTTTTC | *icaR* MxiE box mutation A16C |
| HMIO271  HMIO272 | ATCAACAATACCAGCGCAGCTTAC  CATGATTTTATTCCATGTTGATTGATTTGTTTCC | 5’ deletion of the first 15 bp of *icaT* |
| HMIO273  HMIO272 | GCAGCTTACCCAGAATCCATCA  CATGATTTTATTCCATGTTGATTGATTTGTTTCC | 5’ deletion of the first 30 bp of *icaT* |
| HMIO274  HMIO272 | AACAATGATGAAATTAATGGATTAGTACAAGAGTTCA  CATGATTTTATTCCATGTTGATTGATTTGTTTCC | 5’ deletion of the first 60 bp of *icaT* |
| HMIO276  HMIO275 | TTGAATTATTCTCAACACATTACATTAGC  CATGACGAACTCCTTGAGTTAATAAGAAC | 5’ deletion of the first 15 bp of *icaR* |
| HMIO277  HMIO275 | CACATTACATTAGCCGACAATTTTAAACA  CATGACGAACTCCTTGAGTTAATAAGAAC | 5’ deletion of the first 30 bp of *icaR* |
| HMIO278  HMIO275 | AAAAATGAAGCCTTAGATACCTGGTATGTG  CATGACGAACTCCTTGAGTTAATAAGAAC | 5’ deletion of the first 60 bp of *icaR* |
| ddO1  ddO2 | ACTCAGACATACGGTAACGGAA  ACAATAGCGAGAGAGTCG | ddPCR of *icaT* |
| ddO7  ddO8 | CAACACATTACATTAGCCGACA  CTTCGTTGGAGCGTTTGCTA | ddPCR of *icaR* |
| **Primer** | **Sequence (5’-3’)** | **Description** |
| qPCR87  qPCR88 | ATCTACGGACCGGAATCTT  GATATCGACGCCCAGTTTAC | ddPCR of *recA* |
| HMIO546  HMIO547 | CCCGGGGGTGGCGGATCC  GAATTCGTAATCTTAGCTAGTTACTCGAGGTCATAG | Amplification of plasmid pSU2.1tt-TEM3-M182T (20) |
| HMIO548  HMIO549 | CTAGCTAAGATTACGAATTCACGTTCTTATTAACTCAAGGAG  TGGGATCCGCCACCCCCGGGCCCTTTATTCAATGCTTTTTCTAATAAATC | Amplification of *icaR* with his endogenous SD from pNS9 |
| HMIO550  HMIO551 | CTAGCTAAGATTACGAATTCAACAAATCAATCAACATGGAATAAAATC  TGGGATCCGCCACCCCCGGGAATATCATCAAAATTAACACCTAGTG | Amplification of *icaT* with his endogenous SD from pNS10 |
| HMIO574  HMIO578 | CCCGGGGGTGGCGGATCC  TTGTTTAAAATTGTCGGCTAATGTAATGTGTTGAG | Construction of *icaR* 1-60 bp |
| HMIO276  HMIO582 | TTGAATTATTCTCAACACATTACATTAGC  CATGACGAACTCCTTGAGTTAATAAG | 5’ deletion of the first 15 bp of *icaR* |
| HMIO574  HMIO576 | CCCGGGGGTGGCGGATCC  ATTTTCATTGATGGATTCTGGGTAAGCTGC | Construction of  *icaT* 1-60 bp |
| HMIO271  HMIO580 | ATCAACAATACCAGCGCAGCTTAC  CATGATTTTATTCCATGTTGATTGATTTG | 5’ deletion of the first 15 bp of *icaT* |
| HMIO595  HMIO596 | TTCTAGGAGGTGCTAGCGCTATGATTGAAGAAGGGCTGTTAC  AGTTTCTGTTCCTCGAGCCCTAACTTTTTACATAGTTCTCTCGG | Amplification of *eivF* from ATCC43888 for Gibson cloning |
| HMIO605  HMIO606 | TTCGCTAGGAGAAATTAACCATGGACACAGAAACAATTG AAATATTC  TGATCTTTATAATCGGATCCGCCATTATCTTCTGAATTA TCGG | Amplification of *ygeG* from ATCC43888  for Gibson cloning |
| HMIO593  HMIO594 | GGGCTCGAGGAACAGAAAC  AGCGCTAGCACCTCCTAG | Amplification of plasmid pSU2.1 for Gibson cloning |
| HMIO603  HMIO604 | GGATCCGATTATAAAGATCATGAC  GTTAATTTCTCCTAGCGAATTC | Amplification of plasmid pUC18.1  for Gibson cloning |
